# Garcin syndrome caused by sphenoid bone metastasis of lung cancer: a case study

**DOI:** 10.1186/s12957-018-1351-4

**Published:** 2018-03-06

**Authors:** Satoshi Fukai, Naoyuki Okabe, Hayato Mine, Hironori Takagi, Hiroyuki Suzuki

**Affiliations:** 1 Department of Chest Surgery, Takeda General Hospital, 3-27 Yamaga, Aizuwakamatsu, 965-8585 Japan; 2 Department of Chest Surgery, Fukushima Medical University, 1 Hikarigaoka Fukushima, Fukushima, 960-1295 Japan

**Keywords:** Garcin syndrome, Lung cancer, Bone metastasis, Sphenoid bone, Basal bone, Cranial nerve palsy

## Abstract

**Background:**

Garcin syndrome, which consists of unilateral palsies of almost all cranial nerves without either sensory or motor long-tract disturbances or intracranial hypertension, can be caused by malignant tumors at the skull base. The case of a patient with lung cancer that metastasized to the sphenoid bone and resulted in Garcin syndrome is presented.

**Case presentation:**

A 76-year-old woman was diagnosed as having non-small cell lung cancer with pericardial and diaphragmatic infiltration, cT4N1M0, stage 3A. The left lower lobectomy with concomitant resection of the pericardium and diaphragm was performed. The pathological diagnosis was pleomorphic carcinoma, pT2bN0M0, stage 1B. She was then followed in the surgery clinic, and 2 months after surgery, she visited an emergency room complaining of headache and diplopia. Neurological examination showed the left IV, V1, and VI cranial nerve palsies. Metastatic tumor with bone destruction was found in the left sphenoid sinus on head computed tomography (CT) and contrast magnetic resonance imaging (MRI), and she was diagnosed with Garcin syndrome caused by sphenoid bone metastasis of lung cancer. Irradiation was performed as palliative treatment, but her neurological findings did not improve. Her general condition gradually worsened, and she died 5 months after surgery.

**Conclusions:**

Bone metastasis of lung cancer occurs frequently, but sphenoid bone metastasis is extremely rare. In this case report, Garcin syndrome caused by lung cancer is discussed in the context of the few previous reports.

## Background

Guillain-Alajouanine-Garcin syndrome (Garcin syndrome) is a rare syndrome first reported by Garcin in 1926 [1]. Garcin syndrome consists of unilateral palsies of almost all cranial nerves without sensory or motor long-tract disturbances or intracranial hypertension that can be caused by malignant tumors at the skull base. In Garcin’s report, it is written as “almost” all cranial nerves, and although the specific number of cranial nerves is not clarified, all of I to XII cranial nerve palsies can occur.

Garcin syndrome could be caused by primary tumors of the skull base, such as nasopharyngeal tumors [1] and epipharyngeal rhabdomyosarcoma [2]; metastatic tumors, such as breast cancer [3], prostate cancer [4], and lung cancer [5–10]; and inflammatory diseases, such as mucormycosis [11]. A case of sphenoid bone metastasis of lung cancer with Garcin syndrome is described.

## Case presentation

**Fig. 1:**
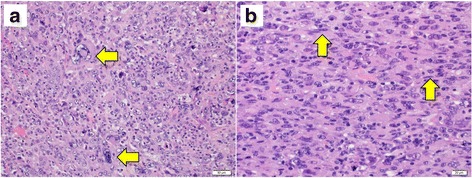
**a** Pathological examination shows pleomorphic carcinoma with giant cells (H&E staining). **b** Many spindle cells are observed (H&E staining)

A 76-year-old woman lost 3 kg in 3 months and visited a primary care clinic in September 2014. She had never smoked, and her Eastern Cooperative Oncology Group (ECOG) performance status (PS) was 1. Computed tomography (CT) showed a tumor of about 6 cm in the left lower lobe. Bronchoscopic biopsy did not show malignant cells, but sarcomatoid cancer was suspected with bronchoalveolar lavage fluid (BALF). Preoperative brain magnetic resonance imaging (MRI) showed no brain metastasis, and metastasis was not found in whole-body positron emission tomography (PET) CT. In contrast CT, the tumor was widely in contact with the pericardium and the diaphragm. After examination, a non-small cell lung cancer with pericardial and diaphragmatic invasion, cT4N1M0, stage3A, was diagnosed. The left lower lobectomy with concomitant resection of the pericardium and diaphragm and nodal dissection (ND)2a-2 was then performed. The pathological diagnosis was pleomorphic carcinoma (Fig. 1), pT2bN0M0, stage 1B. The patient was followed in the surgery clinic without chemotherapy or radiotherapy. Two months after surgery, she visited an emergency room with chief complaints of headache that the throbbing of the left temple makes it impossible to open her eyes and diplopia.

**Fig. 2:**
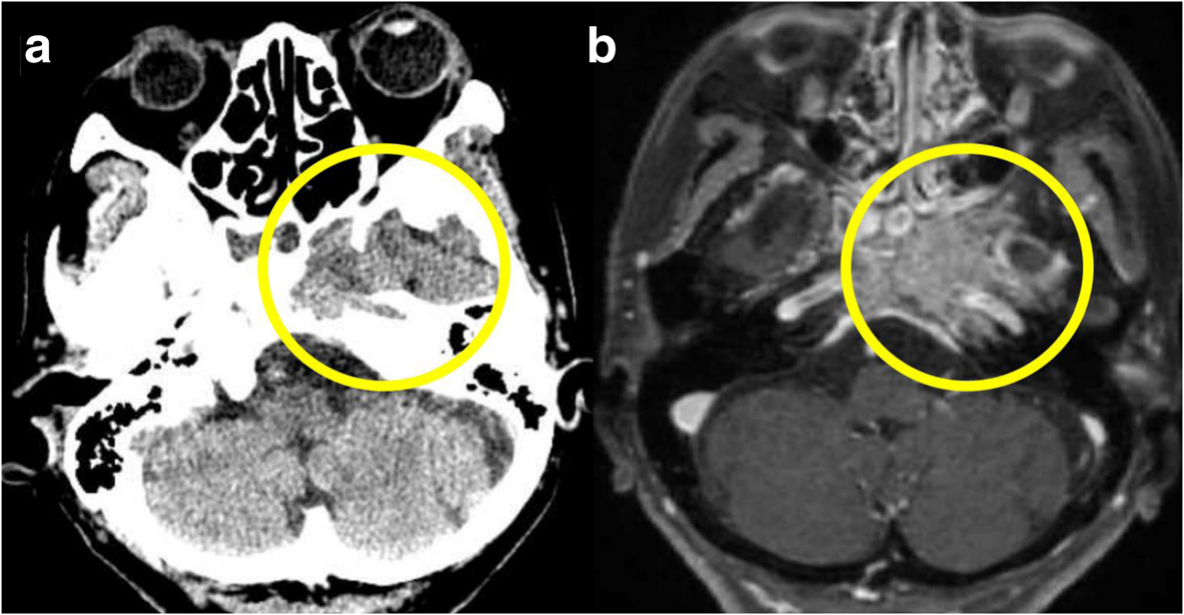
**a** Head CT shows bone destruction at the left sphenoid sinus. **b** Gadolinium-enhanced MRI (T1WI) of the brain shows a contrast-enhanced tumor at the left sphenoid sinus

On neurological examination, she showed left trochlear (IV) and abducens (VI) nerve palsies and had pain in the left trigeminal nerve first branch area (V1). Head CT showed bone destruction at the left sphenoid sinus (Fig. 2a). Gadolinium-enhanced MRI of the brain showed a contrast-enhanced tumor (Fig. 2b). Based on the neurologic examination and imaging findings, Garcin syndrome caused by sphenoid bone metastasis of lung cancer was diagnosed. Five-gray irradiation was performed four times to control her neurological symptoms, but they did not improve. Subsequently, she was given zoledronic acid in the surgery clinic, but it was ineffective. Three months after surgery, whole-body PET and CT showed cancer spread to involve the right ninth rib and the left adrenal gland. Her general condition gradually worsened, and she died in March 2015, about 5 months after surgery.

## Discussion and conclusions

Garcin syndrome is defined as meeting the following four diagnostic criteria: (1) unilateral palsies of the cranial nerves, (2) neither sensory nor motor long-tract disturbance, (3) no intracranial hypertension, and (4) an osteoblastic lesion in the skull base [1]. It has unique clinical symptoms and is a rare syndrome. There are Collet-Sicard syndrome, cavernous sinus syndrome, Tolosa-Hunt syndrome which have similar clinical findings with Garcin syndrome, but these syndromes are paralysis of a certain cranial nerve (e.g., Collet-Sicard is IX to XII) and are defined also by nerve inflammation. Garcin syndrome, as shown, distinguishes it from those syndromes in that it can occur in all cranial nerves and cannot be diagnosed without osteoclastic lesions. The sphenoid sinus is a cavity inside the butterfly bone in the midline of the skull base and is one of the paranasal sinuses. The sphenoid sinus is located in the medial of the superior orbital fissure which the III, IV, V1, and VI cranial nerves pass, and in this case, the cranial nerves were compressed by the tumor. Many reports of Garcin syndrome assume that it originates from a bone invading malignant tumor, such as a tumor of the nucleus of the skull base and nasopharynx or metastatic tumor from a remote organ to the skull. Lung cancer is the third most likely to develop bone metastasis following breast cancer and prostate cancer [12]. The rate of bone metastasis at necropsy of lung cancer is reported to be about 20–50 [13]. Furthermore, the histopathological diagnosis of this case was pleomorphic carcinoma, which is reported to be more likely to cause distant metastasis than other types of non-small cell lung cancer [14]. Skull metastasis of lung cancer are reported to be 5 cases in 611 cases (0.4%) of single bone metastasis of lung cancer [12]. Since skull base bone metastasis is rarer than skull metastasis, the overall incidence of Garcin syndrome caused by lung cancer is estimated to be much less than 0.4%. In the previous reports, there were six cases with Garcin syndrome due to lung cancer, including subtypes that did not satisfy all diagnostic criteria (Table 1) [5–10]. To date, there is no specific therapy for Garcin syndrome. In the present case, radiotherapy and zoledronic acid were given as a treatment for bone metastasis, but there was no neurological improvement. A case of small cell lung cancer with neurological improvement after only carboplatin + etoposide therapy has been reported [10]. There are few cases reporting neurological improvement, but there is a possibility that symptoms can be improved by correctly diagnosing Garcin syndrome.

**Table 1:** Characteristics of cases of lung cancer with Garcin syndrome

No.	Age (years)/sex	Histology	Cranial nerves affected	Type of metastasis	Author, year
1	69/F	Adenocarcinoma	Rt. II, V,·VI Lt. II, III, IV, V, VI	Metastasis to the skull base	Imanishi et al. 1974 [5]
2	52/F	Adenocarcinoma	Rt. X, XI, XII	Metastasis to the skull base	Fujii et al. 2007 [6]
3	60/F	Adenocarcinoma	Rt. VII, VIII	Metastasis to the skull base	Toh et al. 2007 [7]
4	50/M	Adenocarcinoma	Lt. V, VII, VIII, IX, X, XII	Cancerous meningitis	Aida et al. 2010 [8]
5	65/F	Non-small cell carcinoma	Rt. III, IV, VII Lt. IX, X	Cancer invasion to the dura mater	Nagashima et al. 2011 [9]
6	61/F	Small cell carcinoma	Lt. IX, XI, XII	Metastasis to the left posterior cranial fossa	Moriyama et al. 2013 [10]
Present case	76/F	Pleomorphic carcinoma	Rt. IV, V1, VI	Metastasis to the sphenoid bone	

A case of recurrence of lung cancer resulting in sphenoid bone metastasis and Garcin syndrome was described. If a patient with lung cancer develops strange neurological manifestations, Garcin syndrome with skull base metastasis must be considered, even though it is an uncommon syndrome. If Garcin syndrome is suspected, it is necessary first to perform contrast MRI and to consult with neurology to clarify neurological findings. Although there is no treatment to be done in an emergency, neurosurgery and radiotherapy are considered next depending on the cause of the disease.

## Abbreviations

BALF: Bronchoalveolar lavage fluid
CT: Computed tomography
ECOG PS: Eastern Cooperative Oncology Group performance status
MRI: Magnetic resonance imaging
ND: Nodal dissection
PET: Positron emission tomography
